# Three Vegetable Laxatives from Trinidad

**Published:** 1909-05-01

**Authors:** 


### Therapeutics and Pharmacy.

### THREE VEGETABLE LAXATIVES FROM TRINIDAD.

The *Journal of Physiology* for February 1908 contains an interesting account by Mr. J. Theodore Cash of his investigations upon the physiological action of certain seeds that were sent by Mr. J. H. Hart, of the Botanical Department, Trinidad, to Kew Gardens, and placed in Mr. Cash's hands for experimental research at the direction of Sir W. Thiselton-Dyer. The original paper must be consulted for details of the experiments that were made upon animals and man. The results seem likely to be of considerable therapeutical service.

The seeds investigated were of three kinds, namely, those of an Euphorbiaceous plant named *Garcia nutans*, and those of two Omphaleas—the *Omphalea triandra*, or Jamaican "cob-nut," and the *Omphalea megacarpa* or *diandra*. In the fresh state these are known in Jamaica to have a laxative action, for which they are sometimes used dietetically.

An oil is obtainable from each of the above varieties of seeds; the actions both of the crude drugs and of the oils were investigated, and Mr. Cash's results are summarised in his paper as follows:

1. The seeds of *Garcia nutans*, *Omphalea triandra*, and *Omphalea diandra* (*megacarpa*) are possessed of purgative properties.

2. The action of the Omphaleas (apart from mechanical effect) appears to be entirely due to the presence of a fixed oil, which, though purgative, is bland and unirritating when given in effective doses. The degree of action of the fresh seeds stands in direct relation to their oily contents.

3. The oils show little tendency to vary with time from their original condition and activity.

4. Garcia nutans seed, whilst containing a purgative oil, possesses an action in excess of the oily contents. It is probable that another (possibly deleterious) principle is contained in the seed, which may belong to the group of toxalbumoses.

5. The oil rapidly undergoes changes in condition and likewise in activity.

6. Garcia nutans and probably also the two omphalea seeds increase peristalsis by stimulating the intramural nervous plexuses (Auerbach's and Meissner's) of the intestine. The intestinal juice is markedly increased by the first, probably by the others to a much slighter extent. The dose of the oils of the omphaleas is sufficient to produce a feeble mechanical action, contributory to the purgative effect which is proper to these oils.

All three seeds produce diuresis, absorption of their principles being thereby indicated. Garcia nutans is the most active in this respect; the omphaleas less so. This action is not exerted through the blood-pressure, as the omphaleas do not affect it, and garcia nutans causes no elevation, but the reverse after it has acted for some time; it is presumable that the tissue of the kidney is directly stimulated, but the exact production of this diuretic action is undetermined.

7. As effective non-irritant cathartics the seed of omphalea triandra and omphalea diandra or their expressed oils would constitute valuable medicinal agencies. The dose sufficient to prove effective is small in bulk, relatively to castor oil, and the taste is far from unpleasant.

Garcia nutans seed causes a prompt effect of a drastic character when given in large dose, but a simple purgative or laxative effect may be developed by modifying the dosage. The oil may probably be found the better and safer agent in the latter capacities.

Some of Mr. Cash's experiments, particularly those carried out in investigating the effects of the seeds and their oils in man, afford some idea of the doses that might be employed in actual therapeutics in practice.

Six minims (0.4 gram) of the oil obtained from garcia nutans seed caused no discomfort, but six hours after ingestion there was a single soft evacuation of the bowels, and there was a similar effect upon the following morning. This dose was repeated on three occasions with very similar results; three times out of four there was some sensation of warmth in the right iliac region between four and five hours after the drug was administered.

Three minim (0.2 gram) doses produced no result on two occasions; on a third there was a single, soft evacuation fifteen and a half hours later. One and a half minim (0.1 gram) doses were invariably without effect. One judges, therefore, that the dose of the drug should be 6 minims or more.

No nausea nor any local irritant action was induced by ingestion of the oil obtained from the omphalea triandra seeds. There was, however, a distinct laxative effect, apparently identical with that produced by the fresh seed. No drastic effect was recorded from doses up to 1 drachm (4 c.c.). Some diuresis without vesical irritation also occurred. A slight laxative effect usually, though not quite invariably, followed a dose of 18 minims (1.2 c.c.), a soft evacuation resulting on the following morning. The effective dose appears to be from 30 to 45 minims (2 to 3 c.c.) of the oil.

The action of the oil obtained from omphalea diandra seeds is closely similar to that of the fresh seed taken in proportion to its oily contents. The kernel yields 64 per cent. of the oil, so that a dose of 1½ drachm (6 grams) of the former, which is known to be distinctly laxative, would furnish about 1 drachm (3.8 grams) of the latter. From this amount of the oil a fairly parallel action results, though, if anything, the effect is slightly below that of 1½ drachm (6 grams) of fresh seed in activity. This may be due to the fresh seed containing less than the usual percentage of oil, and also to the probability that small but irregular fragments may cause an additional mechanical effect favourable to peristalsis, when the seed has been chewed and swallowed. The oil of omphalea diandra is so similar in character as well as in activity to that of omphalea triandra that there is a presumption of their being identical. This point is not yet definitely decided.

